# Emerging Sensing Technologies in Consumer Electronics

**DOI:** 10.3390/s21227689

**Published:** 2021-11-19

**Authors:** Yu-Cheng Fan

**Affiliations:** Department of Electronic Engineering, National Taipei University of Technology, Taipei 10608, Taiwan; skystar@ntut.edu.tw; Tel.: +886-930-974-056

This Special Issue is dedicated to aspects of emerging sensing technologies in consumer electronics. Special interest focuses on advanced sensing components, systems and applications. This Special Issue contains fifteen papers that focus on the healthcare sensor systems, automotive sensor applications, sensors and actuators, virtual reality and augmented reality sensing technology, sensing system for network security and analysis. These emerging sensing technologies will provide the future vision and direction of research scholars in the field of Consumer Electronics.

Emerging sensing technologies play an important role in Consumer Electronics. Designing advanced sensor components, systems and algorithms will be able to improve the functions and performance of consumer electronics. One of the main objectives of the Special Issue of IEEE International Conference on Consumer Electronics-Taiwan focuses on novel sensing technologies [1]. We rigorously selected and published the 15 best papers in an influential and important international journal—Sensors. Five important sensor themes were proposed and discussed, including healthcare sensor systems, automotive sensor applications, emerging sensors and actuators, virtual reality and augmented reality sensing technology, sensing system for network security and analysis. These proposed methods have epoch-making significance, which will be explained in detail in the following content. 

## 1. Healthcare Sensor Systems

In [2], H.-C. Chen et al. designed a computer mouse using blowing sensors intended for people with disabilities to replace the conventional hand-controlled mouse [2]. Its main contribution is that it uses microphones to induce small signals through the principle of airflow vibration, and it then converts the received signal into the corresponding pulse width [2]. The co-design of software programming enables various mouse functions to be implemented by the identification of the blowing pulse width of multiple microphones [2]. The proposed tool is evaluated experimentally, and the experimental results show that the average identification rate of the proposed mouse is over 85% [2]. Additionally, compared with the other mouse assistive tools, the proposed mouse has the benefits of low cost and humanized operation [2]. Therefore, the proposed blowing control method can not only improve the life quality of people with disabilities, but also overcome the disadvantages of existing assistive tools [2]. 

The present methods of diagnosing depression are entirely dependent on self-report ratings or clinical interviews [3]. Those traditional methods are subjective, where the individual may or may not be answering genuinely to questions [3]. Nivedhitha Mahendran et al. presented a sensor-assisted weighted average ensemble model for detecting major depressive disorder [3]. The data has been collected using self-report ratings and also using electronic smartwatches [3]. This study aims to develop a weighted average ensemble machine learning model to predict major depressive disorder (MDD) with superior accuracy [3]. The data has been pre-processed and the essential features have been selected using a correlation-based feature selection method [3]. With the selected features, machine learning approaches such as Logistic Regression, Random Forest, and the proposed Weighted Average Ensemble Model are applied [3]. Further, for assessing the performance of the proposed model, the Area under the Receiver Optimization Characteristic Curves has been used [3]. The results demonstrate that the proposed Weighted Average Ensemble model performs with better accuracy than the Logistic Regression and the Random Forest approaches [3].

Besides, in order to achieve an integrated multi-channel pulse driver for advanced portable ultrasonic imaging systems, Chin Hsia et al. performed a single-chip high-voltage integrated actuator for biomedical ultrasound scanners [4]. The high-voltage (HV) pulse driver based on silicon-on-insulator (SOI) technology for biomedical ultrasound actuators and multi-channel portable imaging systems specifically is proposed in this paper [4]. The pulse driver, which receives an external low-voltage drive signal and produces high-voltage pulses with a balanced rising and falling edge, is designed by synthesizing high-speed, capacitor-coupled level-shifters with a high-voltage H-bridge output stage [4]. In addition, an on-chip floating power supply has also been developed to simplify powering the entire system and reduce static power consumption [4]. The electrical and acoustic performance of the integrated eight-channel pulse driver has been verified by using medical-grade ultrasound probes to acquire the transmit/echo signals [4]. The static power required to support the overall system is less than 3.6 mW, and the power consumption of the system during excitation is less than 50 mW per channel [4]. The integrated multi-channel pulse driver can be used in advanced portable ultrasonic imaging systems [4]. 

## 2. Automotive Sensor Applications

In [5], Y.-S. Chen et al. presented an advanced ICTVSS (intelligent cloud-based transportation vehicle surveillance) model for real-time vehicle traffic applications [5]. In order to reduce the incidence of traffic accidents through by advanced real-time technologies is proposed to develop an advanced system of ICTVSS for license plate identification in this work [5]. The identification algorithm was developed from an improved differential algorithm and active contour algorithm of dynamic license identification for smart monitoring to realize for constructing a well-defined smart city [5]. The algorithm performed well in locating multi-license plate and differential methods, removing image noise of license plate, and processing constant-inconstant light source from complex environment cases, and guaranteed effective license plate identification from the benefit of high resolutions of digital cameras [5]. 

Y.-C. Fan et al. [6] proposed an efficient COordinate Rotation DIgital Computer (CORDIC) iteration circuit design for Light Detection and Ranging (LiDAR) sensors [6,7]. A novel CORDIC architecture that achieves the goal of pre-selecting angles and reduces the number of iterations is presented for LiDAR sensors [8]. The value of the trigonometric functions can be found in seven rotations regardless of the number of input N digits [6]. The number of iterations are reduced by more than half [6]. The experimental results show the similarity value to be all 1 and prove that the LiDAR decoded packet results are exactly the same as the ground truth [6]. This design can not only reduce the number of iterations and the computing time, but also reduce the chip area [6]. The paper provides an efficient CORDIC iteration design and solution for LiDAR sensors to reconstruct the point-cloud map for autonomous vehicles [6]. 

## 3. Emerging Sensors and Actuators

A set of methods for the inspection of a working motor in real time was proposed by W. Lin. Chu et al. [3]. The aim was to determine if ball-bearing operation is normal or abnormal and to conduct an inspection in real time [9]. The system consists of motor control and measurement systems [9]. The motor control system provides a set fixed speed, and the measurement system uses an accelerometer to measure the vibration, and the collected signal data are sent to a PC for analysis [9]. W. Lin. Chu et al. designed the details of the decomposition of vibration signals, using discrete wavelet transform (DWT) and computation of the features [10]. It includes the classification of the features after analysis [9]. Two major methods are used for the diagnosis of malfunction, the support vector machines (SVM) and general regression neural networks (GRNN) [9]. For visualization and to input the signals for visualization, they were input into a convolutional neural network (CNN) for further classification, as well as for the comparison of performance and results [9]. Unique experimental processes were established with a particular hardware combination, and a comparison with commonly used methods was made [9]. The results can be used for the design of a real-time motor that bears a diagnostic and malfunction warning system [9]. 

The maximum power point tracking (MPPT) technique is often used in photovoltaic (PV) systems to extract the maximum power in various environmental conditions [11]. In [11], two reinforcement learning-based maximum power point tracking (RL MPPT) methods are proposed by the use of the Q-learning algorithm [11]. One constructs the Q-table and the other adopts the Q-network [11]. These two proposed methods do not require the information of an actual PV module in advance and can track the MPP through offline training in two phases, the learning phase and the tracking phase [11]. From the experimental results, the RL-QT MPPT method performs with smaller oscillation and the RL-QN MPPT method achieves higher average power [11]. Both the reinforcement learning-based Q-table maximum power point tracking (RL-QT MPPT) and the reinforcement learning-based Q-network maximum power point tracking (RL-QN MPPT) methods have smaller ripples and faster tracking speeds when compared with traditional method [11]. 

In addition, C.-Y. Chang et al. considered real-time evaluation of the mechanical performance and residual life of a notching mold using embedded polyvinylidene fluoride (PVDF) sensors and support vector machine (SVM) criteria [12]. The blunt edges of worn molds can cause the edge of the sheet metal to form a burr, which can seriously impede assembly and reduce the efficiency of the resulting motor [12]. The overuse of molds without sufficient maintenance leads to wasted sheet material, whereas excessive maintenance shortens the life of the punch/die plate [12]. Diagnosing the mechanical performance of die molds requires extensive experience and fine-grained sensor data [12]. C.-Y. Chang et al. embedded PVDF films within the mechanical mold of a notching machine to obtain direct measurements of the reaction forces imposed by the punch and developed an automated diagnosis program based on a SVM to characterize the performance of the mechanical mold [12]. The cyber-physical system (CPS) facilitated the real-time monitoring of machinery for preventative maintenance as well as the implementation of early warning alarms [12]. The cloud server used to gather mold-related data also generated data logs for managers [12]. The hyperplane of the CPS-PVDF was calibrated using a variety of parameters pertaining to the edge characteristics of punches [12].

## 4. Virtual Reality and Augmented Reality Sensing Technology

Y.-S. Chang dealt with a mobile augmented reality (MAR) application supporting teaching activities in interior design [13]. The application supports students in learning interior layout design, interior design symbols, and the effects of different design layout decisions [13]. Utilizing the latest AR technology, users can place 3D models of virtual objects, chairs or tables on top of a design layout plan and interact with these on their mobile devices [13]. Students can experience alternative design decisions in real-time and increase the special perception of interior designs [13]. This system fully supports the import of interior deployment layouts and the generation of 3D models based on design artefacts based on typical design layout plan design symbols and allows the user to investigate different design alternatives [13]. The learning results clearly shows that the reference group utilizing MAR technology as a learning aid show a higher learning effectiveness than the control group [13].

Smart glasses are attracting particular attention because they offer convenient features such as hands-free augmented reality (AR) [14]. Since smart glasses directly touch the face and head, the device with high temperature has a detrimental effect on human physical health [14]. K. Matsuhashi considered a thermal network model in a steady state condition and thermal countermeasure methods for thermal management of future smart glasses [14]. It is accomplished by disassembling the state by wearing smart glasses into some parts, creating the equivalent thermal resistance circuit for each part, approximating heat-generating components such as integrated circuits (ICs) to simple physical structures, setting power consumption to the heat sources, and providing heat transfer coefficients of natural convection in air [14]. Results of an experiment using the model show that the temperature of the part near the ear that directly touches the skin can be reduced by 51.4% by distributing heat sources into both sides, 11.1% by placing higher heat-generating components farther from the ear, and 65.3% in comparison with all high conductivity materials by using a combination of low thermal conductivity materials for temples and temple tips and high conductivity materials for rims [14]. 

Besides, K.-F. Lee et al. aimed to perform gaze tracking and point estimation using low-cost head-mounted devices [15]. To provide a cost-effective vision tracking solution, this head-mounted device is combined with a sized endoscope camera, infrared light, and mobile phone; the devices are also implemented via 3D printing to reduce costs [15]. Based on the proposed image pre-processing techniques, the system can efficiently extract and estimate the pupil ellipse from the camera module [15]. A 3D eye model was also developed to effectively locate eye gaze points from extracted eye images [15]. In the experimental results, average accuracy, precision, and recall rates of the proposed system can achieve an average of over 97%, which can demonstrate the efficiency of the proposed system [15]. This study can be widely used in the Internet of Things, virtual reality, assistive devices, and human–computer interaction applications [15]. 

Since general methods predicting personalized saliency maps (PSMs) need a large number of training images, the establishment of a theory using a small number of training images is needed [16]. To tackle this problem, although finding persons who have visual attention similar to that of a target person is effective, all persons have to commonly gaze at many images [16]. Thus, it becomes difficult and unrealistic when considering their burden [16]. In order to solve this problems, Y. Moroto et al. depicted a few-shot personalized saliency prediction based on adaptive image selection considering object and visual attention [16]. The novel adaptive image selection (AIS) scheme that focuses on the relationship between human visual attention and objects in images [16]. AIS focuses on both a diversity of objects in images and a variance of PSMs for the objects [16]. Specifically, AIS selects images so that selected images have various kinds of objects to maintain their diversity [16]. Moreover, AIS guarantees the high variance of PSMs for persons since it represents the regions that many persons commonly gaze at or do not gaze at [16]. The proposed method enables selecting similar users from a small number of images by selecting images that have high diversities and variances [16]. Experimental results show the effectiveness of the personalized saliency prediction including the new image selection scheme [16]. 

## 5. Sensing System for Network Security and Analysis

A new kind of malware called Mirai is spreading like wildfire [17]. Mirai is characterized by targeting Internet of Things (IoT) devices [17]. Since IoT devices are increasing explosively, it is not realistic to manage their vulnerability by human-wave tactics [17]. S. Yamaguchi proposed a new approach that uses a white-hat worm to fight malware [17]. The white-hat worm is an extension of an IoT worm called Hajime and introduces lifespan and secondary infectivity [17]. The white-hat worm model enables us to simulate a battle between the white-hat worm and Mirai [17]. 

Geographical social networks (GSN) is an emerging research area [18]. These applications are also known as location-based services (LBS) [18]. Previous studies have suggested that these location-based services may expose user location information [18]. In order to ensure the privacy of the user’s location data, the service provider may provide corresponding protection mechanisms for its applications, including spatial cloaking, fuzzy location information, etc., so that the user’s real location cannot be easily cracked [18]. It has been shown that if the positioning data provided by the user is not accurate enough, it is still difficult for an attacker to obtain the user’s true location [18]. T.-L. Lin et al. developed a location privacy attack based on the location sharing mechanism with erroneous distance in geosocial networks [18]. Taking this factor into consideration, the attack method is divided into two stages for the entire attack process [18]: (1) Search stage: cover the area where the targeted user is located with unit discs, and then calculate the minimum dominating set [18]. Use the triangle positioning method to find the minimum precision disc [18]. (2) Inference phase: Considering the existence of errors, an Error-Adjusted Space Partition Attack Algorithm (EASPAA) was proposed during the inference phase [18]. This improved the need for accurate distance information to be able to derive the user’s true location [18]. This study focuses on the Location Sharing Mechanism with Maximal Coverage Limit to implement the whole attack [18]. Experimental results show that the proposed method can still accurately infer the user’s real location even when there is an error in the user’s location information [18].

Additionally, despite advancements in the Internet of Things (IoT) and social networks, developing an intelligent service discovery and composition framework in the Social IoT (SIoT) domain remains a challenge [19]. In the IoT, a large number of things are connected together according to the different objectives of their owners [19]. Due to this extensive connection of heterogeneous objects, generating a suitable recommendation for users becomes very difficult [19]. For the aforementioned reasons, G.-X. Lye et al. presented an SIoT architecture with a personalized recommendation framework to enhance service discovery and composition [19]. This study developed a unique personalized recommender engine that is based on the knowledge-desire-intention model and is suitable for service discovery in a smart community [19]. The algorithm provides service recommendations with high satisfaction by analyzing data concerning users’ beliefs and surroundings [19]. Moreover, the algorithm eliminates the prevalent cold start problem in the early stage of recommendation generation [19]. Several experiments and benchmarking on different datasets are conducted to investigate the performance of the proposed personalized recommender engine [19]. The experimental precision and recall results indicate that the proposed approach can achieve up to an approximately 28% higher F-score than conventional approaches [19]. 

As the applications of consumer electronics become more diversified, emerging sensing technologies becomes more important. The design of sensing components, sensing algorithms, sensing architectures and sensing systems will become the core part of consumer electronics. This Special Issue combines healthcare sensor systems, automotive sensor applications, sensors and actuators, virtual reality and augmented reality sensing technology, sensing system for network security and analysis, and proposes key technologies and methods. The diversity of applications in this Special Issue demonstrates the importance of novel research on emerging sensing technologies for consumer electronics.
